# Multiparametric Tissue Characterization Utilizing the Cellular Metallome and Immuno-Mass Spectrometry Imaging

**DOI:** 10.1021/jacsau.2c00571

**Published:** 2023-02-08

**Authors:** Martin Schaier, Sarah Theiner, Dina Baier, Gabriel Braun, Walter Berger, Gunda Koellensperger

**Affiliations:** † Institute of Analytical Chemistry, Faculty of Chemistry, University of Vienna, Waehringer Strasse 38, Vienna 1090, Austria; ‡ Vienna Doctoral School in Chemistry (DoSChem), University of Vienna, Waehringer Strasse 42, Vienna 1090, Austria; § Institute of Inorganic Chemistry, Faculty of Chemistry, University of Vienna, Waehringer Strasse 42, Vienna 1090, Austria; ∥ Institute of Cancer Research and Comprehensive Cancer Center, Medical University of Vienna, Borschkegasse 8A, Vienna 1090, Austria

**Keywords:** metallome, immune-histochemistry, multiparametric tissue profiling, bioimaging, laser ablation, mass spectrometry

## Abstract

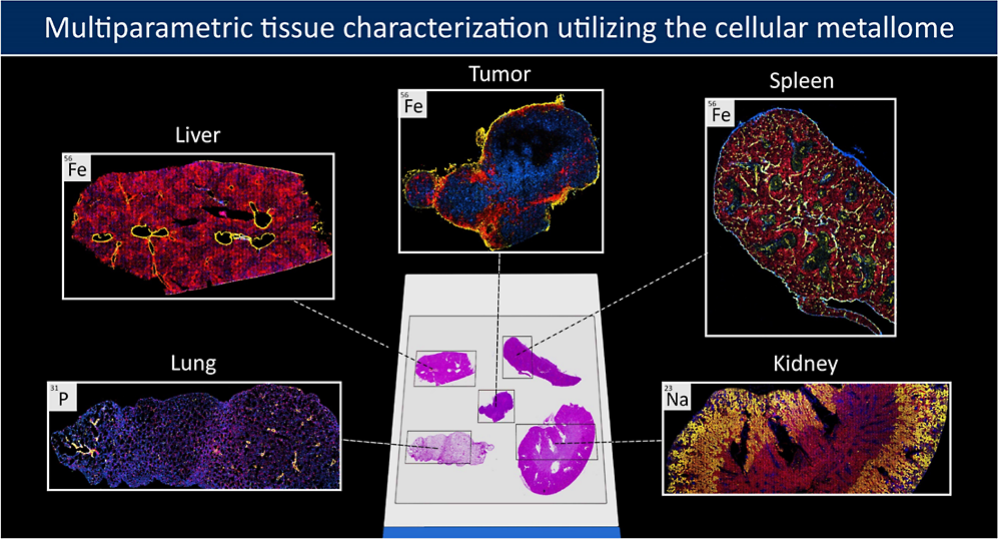

In this study, we present a workflow that enables spatial single-cell metallomics in tissue decoding the cellular heterogeneity. Low-dispersion laser ablation in combination with inductively coupled plasma time-of-flight mass spectrometry (LA-ICP-TOFMS) provides mapping of endogenous elements with cellular resolution at unprecedented speed. Capturing the heterogeneity of the cellular population by metals only is of limited use as the cell type, functionality, and cell state remain elusive. Therefore, we expanded the toolbox of single-cell metallomics by integrating the concepts of imaging mass cytometry (IMC). This multiparametric assay successfully utilizes metal-labeled antibodies for cellular tissue profiling. One important challenge is the need to preserve the original metallome in the sample upon immunostaining. Therefore, we studied the impact of extensive labeling on the obtained endogenous cellular ionome data by quantifying elemental levels in consecutive tissue sections (with and without immunostaining) and correlating elements with structural markers and histological features. Our experiments showed that the elemental tissue distribution remained intact for selected elements such as sodium, phosphorus, and iron, while absolute quantification was precluded. We hypothesize that this integrated assay not only advances single-cell metallomics (enabling to link metal accumulation to multi-dimensional characterization of cells/cell populations), but in turn also enhances selectivity in IMC, as in selected cases, labeling strategies can be validated by elemental data. We showcase the power of this integrated single-cell toolbox using an in vivo tumor model in mice and provide mapping of the sodium and iron homeostasis as linked to different cell types and function in mouse organs (such as spleen, kidney, and liver). Phosphorus distribution maps added structural information, paralleled by the DNA intercalator visualizing the cellular nuclei. Overall, iron imaging was the most relevant addition to IMC. In tumor samples, for example, iron-rich regions correlated with high proliferation and/or located blood vessels, which are key for potential drug delivery.

## Introduction

Studying the metallome in biological samples at the cellular level by different imaging techniques has become important due to the crucial role of endogenous metals in metal homeostasis and as a consequence, in the context of diseases.^1^ While bulk elements (e.g., Na, K, and Mg) are essential for structure and information transfer in the body, trace metals (e.g., Fe, Cu, and Zn) form metalloproteins with catalytic function, like the superoxide dismutase (SOD), which is involved in the removal of free radicals.^2^ Even minor changes in metal homeostasis can indicate disease development, with the well-known examples of Menkes or Wilson’s disease, where copper transport is disturbed by genetic defects.^3^ The former is characterized by a copper deficiency, leading to progressive neurodegeneration, while in the latter, an excess of copper can cause cellular damage.^4,5^

Laser ablation–inductively coupled plasma mass spectrometry (LA-ICPMS) has become an established technique for multi-element mapping of biological samples, providing high sensitivity, high sample throughput, and spatial resolutions in the low μm range.^6−8^ Bioimaging applications by LA-ICPMS are primarily dominated by mapping of the metallome in various types of tissue samples (e.g., in the context of neurodegenerative diseases) and by studying the uptake of metallodrugs and nanoparticles in biological systems. The newest low-dispersion LA setups have enabled (sub-)cellular imaging (with spot sizes down to 1 μm) and the analysis of single cells at pixel acquisition rates of >200 Hz.^9−11^ These technological advancements of LA-ICPMS techniques have evolved to the concept of imaging mass cytometry (IMC),^12^ where phenotyping of single cells is performed in tissue samples with a spot size of 1 μm and pixel acquisition rates of 200 Hz (400 Hz with the latest instrumental generation). Highly multiplexed immunohistochemistry studies can be performed using antibodies that are labeled with different metal tags followed by LA-ICP-TOFMS detection. Metal tags include MaxPar metal-conjugated reagents, MeCATs (metal-coded affinity tags), single-atom chelates, Au/Ag nanoparticles, fluorescent Au nanoclusters, and quantum dots.^13−18^ In pioneering studies by Wang et al.^19^ and Giesen et al.,^20^ highly multiplexed imaging of epidermal growth factor receptor 2 and 32 proteins was performed in human breast tissue sections. The CyTOF equipment designed for IMC analysis has been targeted toward the clinical field, with a growing field of applications in cancer research, immune-profiling, neurodegenerative diseases, and so forth.^21−26^ The latest generation of CyTOF instrumentation provides the detection of *m*/*z* = 75–209, as it was specifically designed for lanthanide detection. As a drawback, the analysis of elements from the lower mass range with biological key functions is not possible with this kind of instrumentation. There are currently different ICP-TOFMS systems on the market that provide the capability to measure *m*/*z* = 14–256^27−29^ and, therefore, imaging of endogenous elements at the cellular level.^6^ Several LA-ICPMS studies employed quadrupole-based ICP–MS systems, limiting the number of elements that can be measured simultaneously. Up to now, only a few imaging studies have addressed the combined detection of endogenous elements and metal-conjugated antibodies by LA-ICP-QMS. The assessment of co-localization of proteins with endogenous metals offers the possibility for studying the interactions between proteins and metal cofactors. In this regard, LA-ICP-QMS studies examined the relationship between nanoparticle/metal-tagged tyrosine hydroxylase (which served as proxy for dopamine) and iron levels in a mouse model of Parkinson’s disease.^16,30,31^ Co-localization of high Fe and dopamine levels was observed in the substantia nigra pars compacta and in the hypothalamus, in which dysfunction is associated with non-motor symptoms of Parkinson’s disease.^16,31^

With regard to sample preparation protocols for tissue imaging by LA-ICPMS, cryo-sectioning and formalin fixation followed by paraffin embedding (FFPE) or resin embedding are the most common ones. The effect of these methods on the spatial distribution of elements and their quantities in tissues was already investigated by LA-ICPMS measurements.^32−34^ A significant influence of the FFPE approach was observed for alkaline elements, while transition metals were less affected by the sample preparation steps. Especially, for calcium and zinc, contaminations introduced during the embedding process could be identified.^34^

Currently, there is no systematic study available for assessing the background levels of endogenous elements after multi-step immunostaining protocols. Therefore, this work evaluates the influence of immunostaining on the concentration and distribution of these elements for different tissue types and sample preparation protocols. For this purpose, different tissue sections (cryo-sections and FFPE sections) of mouse organs and tumor were compared by LA-ICP-TOFMS analysis before and after immunostaining. Elemental quantification was carried out using gelatin-based micro-droplet standards.^35,36^ Co-localization with hematoxylin and eosin stains as well as metal-conjugated antibodies was used to further validate the results. Based on these validations, selected elements with biological key functions (Na, P, and Fe) were visualized in different biological applications together with metal-conjugated antibodies to achieve highly multiplexed imaging with cellular resolution. The goal of this combined approach was to reveal different cell types and structural features within biological tissues and to relate them to the elemental homeostasis. In particular, there is great potential for iron, as it is an integral component of many organ functions. By comparing LA-ICP-TOFMS results with histological sections, the added value of this technology for answering biological questions will be demonstrated.

## Results and Discussion

### Impact of Sample Preparation on the Endogenous Elemental Distribution

Formalin fixation and paraffin embedding has emerged as gold standard for histological evaluations in clinics, as it preserves essential tissue structures and proteins, making it the ideal method for long-term storage. Tissue sections that are prepared by FFPE protocols undergo extensive washing and solvent treatment, which can significantly alter the qualitative and quantitative distributions of endogenous elements such as iron, copper, and zinc.^34^ An alternative is cryo-sectioning, where the tissue is directly frozen in an embedding matrix without any further steps prior to sectioning. Due to the reduced number of sample preparation steps, it is the preferred method for the LA-ICPMS analysis of intrinsic elements in biological samples. Sample preparation involving tissue embedding and/or immunostaining, where the tissue is exposed to multiple chemicals and washing steps can contribute to potential elemental contaminations and wash-out effects.

Therefore, we systematically evaluated the effect of different sample preparation protocols, followed by immunostaining on elements intrinsically present in biological samples. Absolute quantification was achieved by multi-level matrix-matching calibrations established by gelatin micro-droplets, where low fg/pixel concentration levels mark the lower limits of quantification. Typically, the endogenous tissue/cellular concentration of, for example, sodium, magnesium, phosphorus iron, copper, and zinc, fall within the working range of the quantification method.

Quantitative LA-ICP-TOFMS analysis of consecutive tumor tissue sections (cryo-sections and FFPE sections) revealed significant alterations of elemental levels for Mg, K, Ca, Cu, and Zn and, to a lesser extent, for Na, P, and Fe upon immuno-labeling (Figure S1, Tables S1 and S2). The observed impact was comparable for cryo-sections and FFPE sections, with the exception of Na and Fe. While Na and Fe levels decreased in cryo-sections upon labeling, the same procedure resulted in increased levels in FFPE tissue. P showed a consistent loss of up to 40% for both sample preparation types after the immunostaining. However, no significant changes in qualitative elemental distributions were observed for these three elements.

Highly elevated Cu concentrations were observed after the labeling procedure, most likely resulting from impurities of the TBS wash solution and due to high physiological Cu levels of BSA used during the blocking step. For Zn, a signal loss of almost 90% was observed in the different tissue samples after the immunostaining approach. For both elements, the qualitative distribution pattern also changed, which makes their detection in stained tissue sections unreliable. The labeling procedure also had a significant impact on the signal intensities of Mg, K, and Ca, with a signal loss of up to 99%, resulting in signals under the limit of detection for these elements after labeling.

Overall, it was concluded that accurate absolute quantification of endogenous elements present in biological samples is precluded, when immunostaining protocols are applied. This applies to FFPE and cryo-sections, despite the fact that for cryo-sectioning, the number of sample preparation steps is reduced to a minimum. Indeed, sections without antibody labeling would still be required to obtain absolute quantitative results on endogenous elements. We focused our investigation on FFPE tissue due to the following reasons: (i) the cell morphology is better preserved in FFPE tissue sections (Figure S2), an important factor for single-cell analysis and cell segmentation; (ii) cryo-sections tend to show more cutting artefacts than FFPE sections, which can result in tissue folding and cell overlap (Figure S2); (iii) only a fraction of commercially available metal-conjugated antibodies can be used on cryo-sections.

### Co-localization with Histological Features and Metal-Conjugated Antibodies

**Figure 1:**
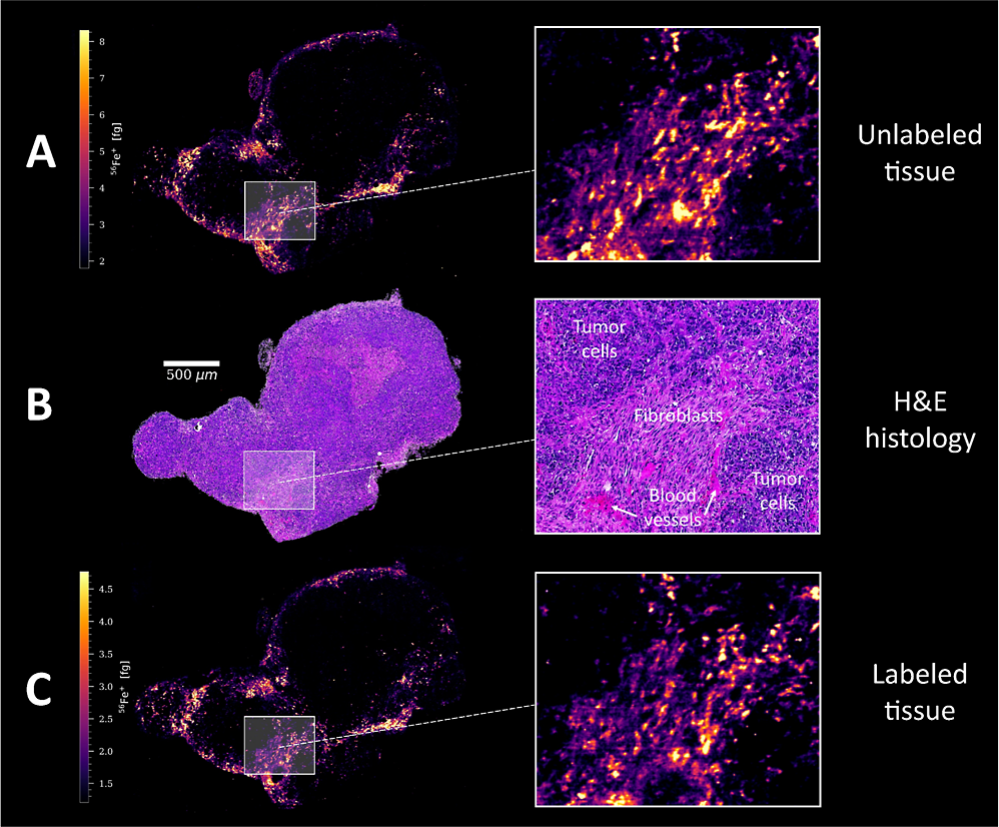
Signal intensity maps of ^56^Fe^+^ in mouse tumor of consecutive FFPE sections (A) before and (C) after the metal-conjugated antibody staining procedure, as determined by LA-ICP-TOFMS analysis. (B) H&E-stained tumor tissue of an adjacent FFPE section for microscopic evaluation.

**Figure 2:**
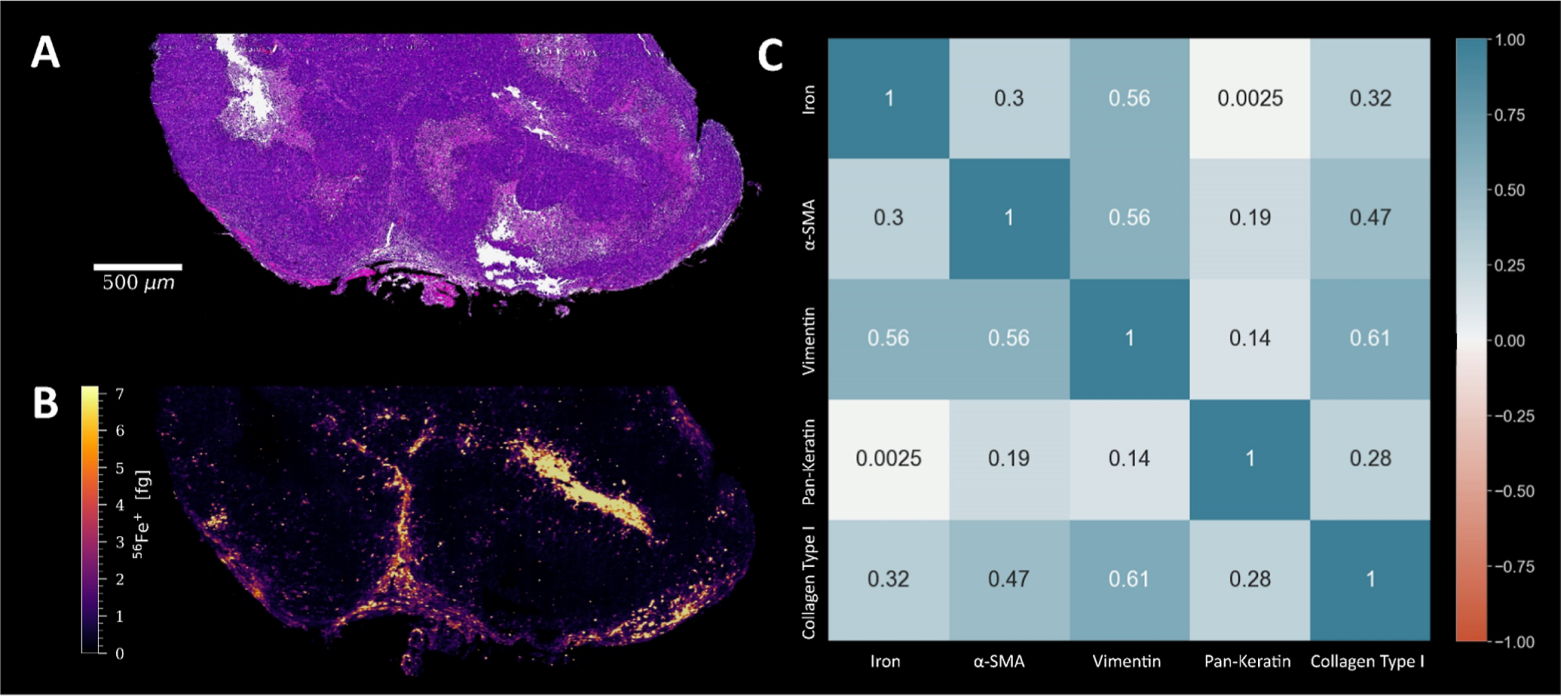
(A) FFPE section of a mouse tumor tissue stained with H&E. (B) Corresponding ^56^Fe^+^ signal intensity map of a consecutive tumor section, determined by LA-ICP-TOFMS analysis. (C) Co-localization of the iron signal was assessed using a correlation matrix with four different antibodies. The values show Pearson correlation coefficients (0.5–1: strong positive, 0.3–0.5: moderate positive, 0–0.3: weak positive).

With regard to the qualitative distribution of endogenous elements after multi-step sample preparation protocols, an orthogonal method such as the microscopic evaluation of a histological stain of an adjacent section is required to obtain reliable information. It has to be considered that consecutive sections are not identical and that changes in the tissue structure can occur, specifically at the single-cell level. Co-localization of the endogenous elemental pattern with distinct histological features and/or with tissue structures/cell types as visualized by metal-conjugated antibodies enables the use of endogenous elements as an additional layer of information in LA-ICP-TOFMS images. As a first step of validation, the similarity of the iron signal intensity maps of consecutive tumor tissue sections (Figure 1) was evaluated using the Structural Similarity Index (SSIM), which compares image parameters such as luminance, contrast, and structure.^37^ A score of 0.82 was obtained (Figure S3), which indicates strong similarity (1 → very strong, −1 → very weak), especially considering that the sections were consecutive and already showed structural differences during histological evaluation (Figure 1). The correlation matrix showed significant co-localization of iron with vimentin (a marker for mesenchymal cells including fibroblasts and endothelial cells of blood vessels) and also to a lower extent with α-SMA (myofibroblasts) and collagen, which are all integral parts of the connective tissue (Figure 2). Hardly any correlation was observed for pan-keratin, which marks the epithelial cells in the tumor. Furthermore, by selecting different regions of interest (ROIs), it could be determined that iron showed the highest correlation with vimentin within the tumor, while in the outer regions of the tumor tissue, iron showed the highest correlation with α-SMA and collagen (Figure S4). The iron distribution can therefore be assigned to biological characteristics of the tumor microenvironment both visually (via an H&E stain and SSIM) and statistically (via correlation with metal-conjugated antibodies).

In a next step, selected applications of LA-ICP-TOFMS imaging in different mouse tissue samples will be presented, highlighting the combined analysis of elements with biological key functions and metal-conjugated antibodies for visualization of characteristic tissue structures and cell types.

### Applications for the Measurement of Endogenous Elements and Metal-Conjugated Antibodies

#### Spleen

**Figure 3:**
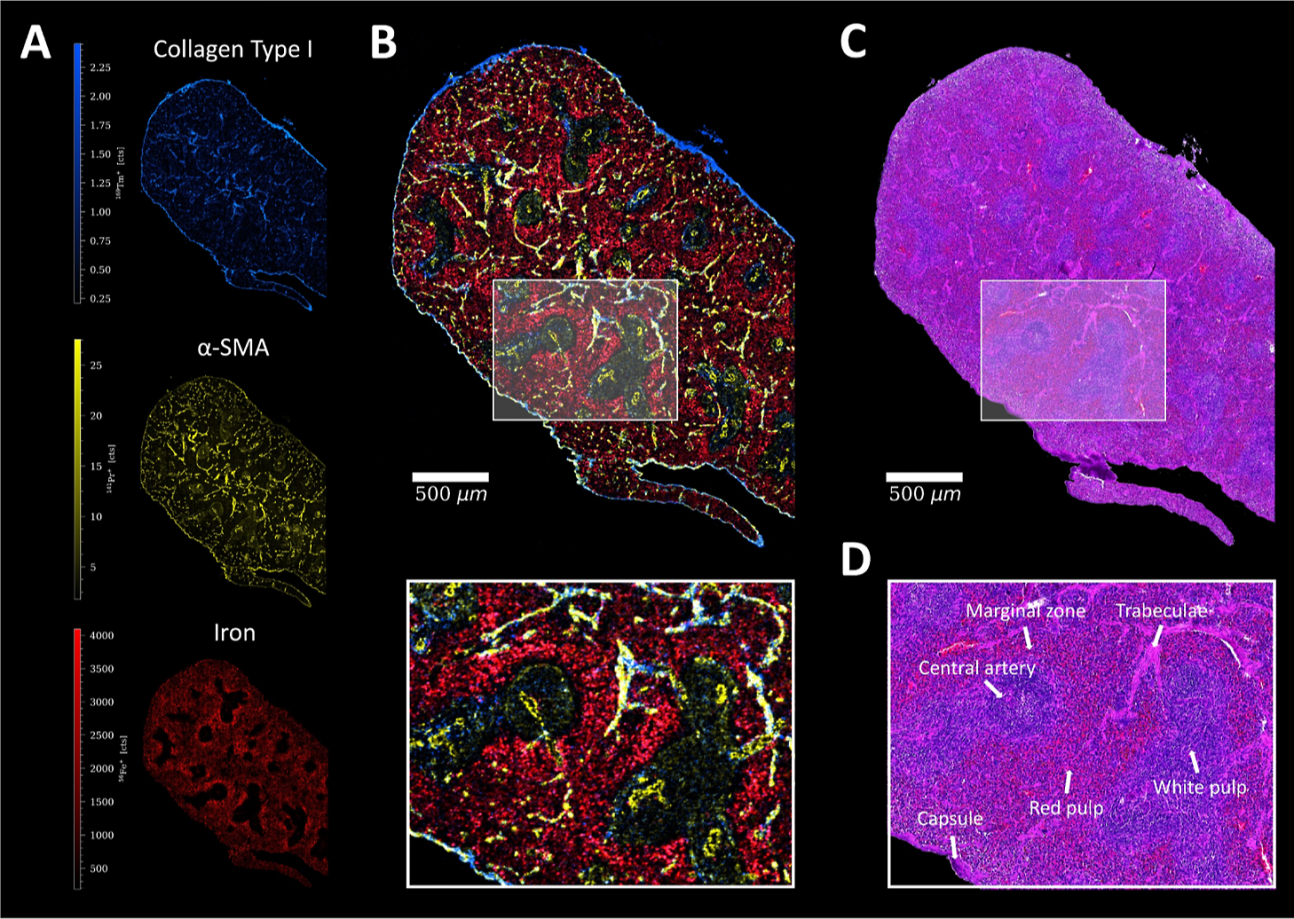
(A) Signal intensity maps and (B) signal overlay of Collagen Type I (blue), α-SMA (yellow), and ^56^Fe^+^ (red) in the spleen of a mouse, with a ROI. (C) Corresponding H&E stain of a consecutive spleen section and (D) ROI with characteristic histological features.

In the spleen, α-SMA and collagen enabled visualization of the splenic capsule (dense collagenous tissue with smooth muscle cells surrounding the spleen) and trabeculae, which are projections from the capsule into the parenchyma containing arteries and veins (Figure 3). The iron distribution as determined by LA-ICP-TOFMS imaging enabled to differentiate the white and red pulp and correlated with histological features, where high amounts of blood were present and circulating. High iron levels were detected in the red pulp, which is known to be responsible for blood filtering in the spleen, whereas a relatively low iron signal was found in the white pulp (Figure 3).^38^ The well-perfused red pulp also showed increased signal levels of KI-67, indicating high levels of proliferation (Figure S5). The highest iron content was observed in the marginal zone (interface between white and red pulp), which is known to exhibit a high blood circulation.^39^ The iron-rich marginal zone also showed higher intensities of CD19 (marker for B-cells) and Arginase-1 (M2 macrophages), while CD86 (M1 macrophages) was expressed to a higher extent in the other regions of the red pulp (Figure S5). These results are in good accordance with the literature, where a high number of B-cells and macrophages was reported in the marginal zone and the red pulps of the spleen, respectively.^40−42^ However, since the spleen was derived from an immunodeficient mouse model (CB-17/SCID), it has to be mentioned that the B- and T-cell levels were relatively low.

#### Liver

**Figure 4:**
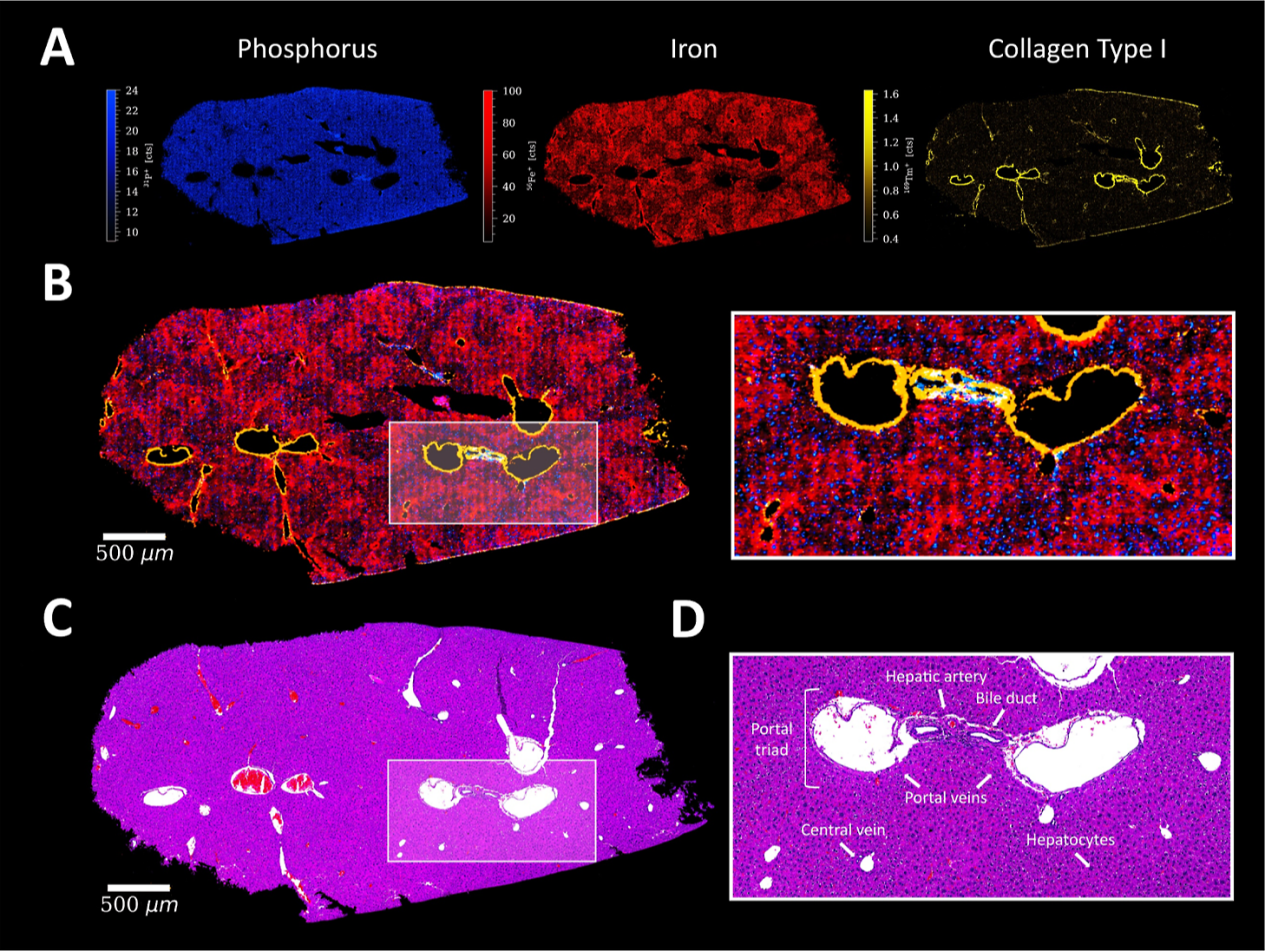
(A) Signal intensity maps and (B) signal overlay of ^31^P^+^ (blue), ^56^Fe^+^ (red), and Collagen Type I (yellow) in liver tissue of a mouse, with a ROI. (C) Corresponding H&E stain of a consecutive liver section and (D) ROI with characteristic histological features.

Within the liver, the iron signal allowed for visualization of the blood flow, starting with low amounts of iron around the portal triad (bile duct, portal vein, and arteriole surrounded by a collagenous matrix) and increasing iron levels around the central veins (Figure 4). Phosphorus can be used to visualize the cell nuclei of individual hepatocytes with a similar signal as the iridium-based DNA intercalator, commonly used for IMC applications (Figure S6). The presence of the central veins in the liver was indicated by Collagen Type I, whereas α-SMA showed a thin layer in the inner side of the portal veins with high intensities around the hepatic artery (Figure S7).^43^ Interestingly, high abundance of E-Cadherin (cell–cell adhesion) was observed in regions with low iron content and seemed to connect the portal vein with the central vein. The apoptosis marker, Caspase 3, also showed a high intensity region in the center of the liver section, located directly on a vein.

#### Kidney

**Figure 5:**
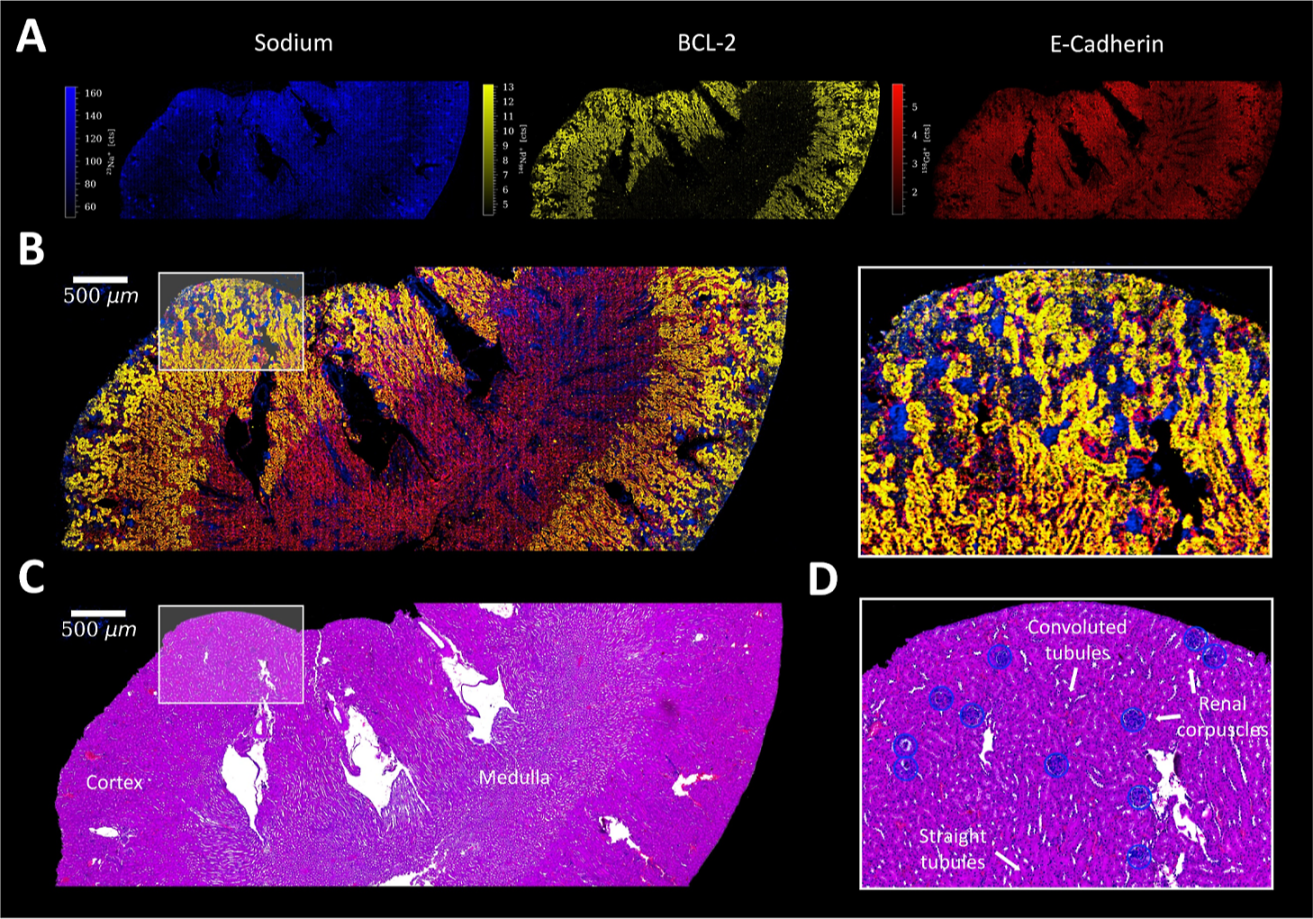
(A) Signal intensity maps and (B) signal overlay of ^23^Na^+^ (blue), BCL-2 (yellow), and E-Cadherin (red) in mouse kidney, with a ROI. (C) Corresponding H&E stain of a consecutive kidney section and (D) ROI with characteristic histological features, which are marked in yellow.

High levels of sodium within the kidney were detected in the proximal convoluted tubules of the cortex region, whereas the proximal straight tubules inside the medulla showed lower sodium intensities (Figure 5). The highest sodium levels were found in the renal corpuscles, where filtering through the glomerular barrier takes place.^44^ Since sodium as an alkali metal is highly soluble and more easily affected by wash-out effects than, for example, P and Fe, it is particularly important to correlate its distribution with histological features and to set it in a biological context. In the investigated case, sodium hotspots could be directly correlated with the presence of renal corpuscles, as observed in the H&E stain of a consecutive kidney section (Figure 5D).

A higher magnification can be seen in Figure S8. The presence of proximal tubules was indicated by the BCL-2 marker, which plays a role in apoptosis regulation and was found to be strongly expressed in proximal convoluted tubules, lower in proximal straight tubules, and weakly in distal tubules (Figure 5).^45^ High sodium intensities were also found inside the collecting ducts of the medullary rays, which are involved in sodium homeostasis by regulating the amounts that get excreted in the urine.^46^ E-Cadherin proved to be more suited for the visualization of the kidney structure than the Ir-based DNA-intercalator since the renal cells contain multiple cell nuclei inside tubules (Figure S9). Using collagen and α-SMA, renal arteries could be visualized (Figure S10). Iron was found predominantly in the proximal tubules of the cortex, but also in medullary rays, which were enriched in pan-keratin.

#### Lung

**Figure 6:**
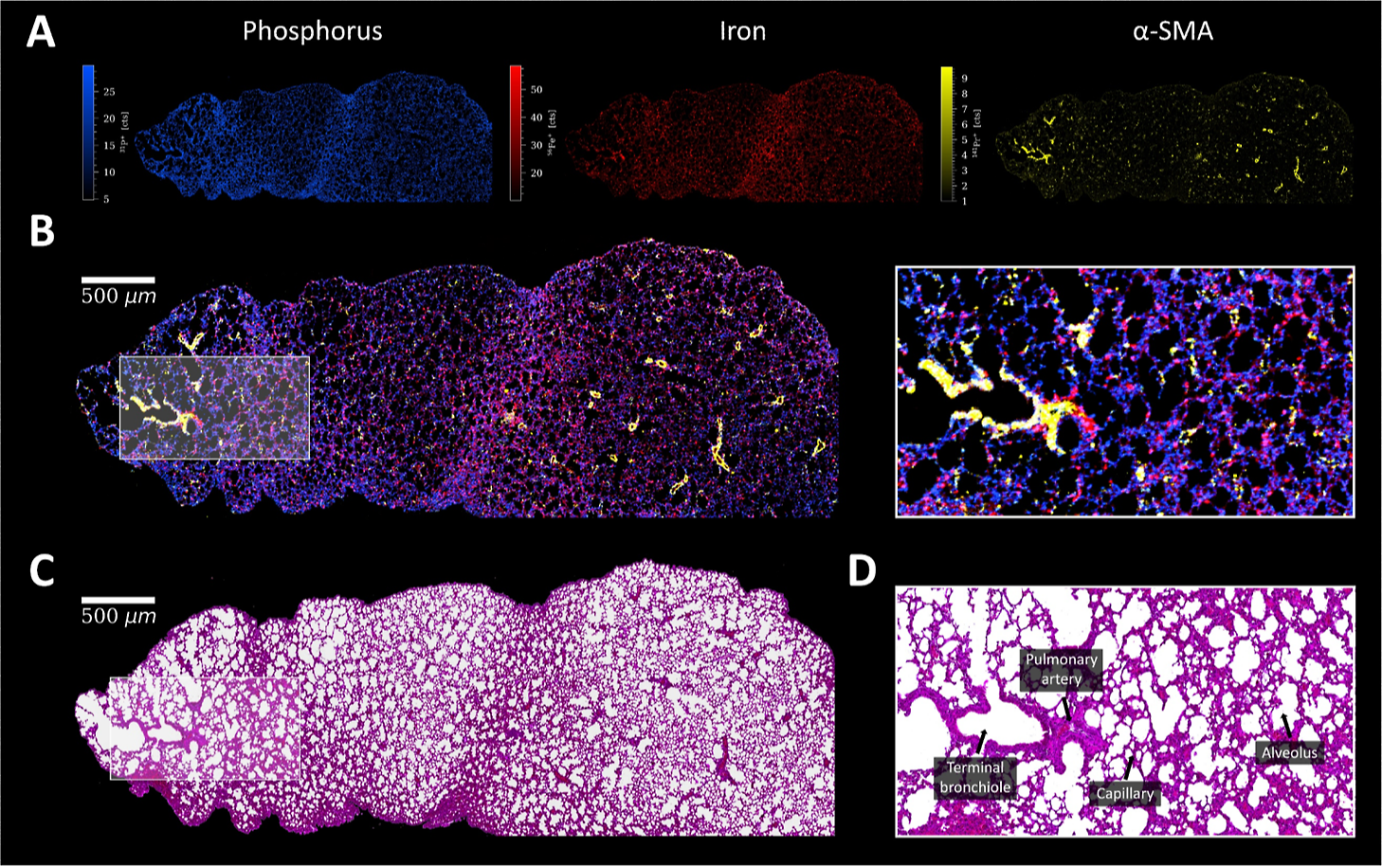
(A) Signal intensity maps and (B) signal overlay of ^31^P^+^ (blue), ^56^Fe^+^ (red), and α-SMA (yellow) in mouse lung, with a ROI. (B) Corresponding H&E stain of a consecutive lung section and (C) ROI with characteristic histological features.

The phosphorus signal provided an overview of the lung structure, showing cell nuclei and cytoplasm, while the iron signal allowed for visualization of the erythrocytes of the capillaries surrounding the alveoli (Figure 6). Blood vessels, terminal bronchiole, and the pulmonary artery were surrounded by α-SMA (Figure 6). Collagen was predominantly found in arteries and outer regions of the lung, while pan-keratin and cadherin were increased in the inner regions of the bronchiole and pulmonary artery (Figure S11). Individual cells within the capillaries showed proliferation (KI-67) with accumulation in one central region. As the organs were taken from a tumor-bearing mouse, this might indicate a metastatic event in the lung, which is further supported by the H&E stain (Figure S12A). The cell lump in this area showed low perfusion and the cell nuclei have a slightly different color. In addition to KI-67, increased intensities of CD44 (cell adhesion) and CD11b (innate immune cell marker) were also found, indicating tumor cell infiltration accompanied by an inflammatory event (Figure S12B).

#### Tumor

**Figure 7:**
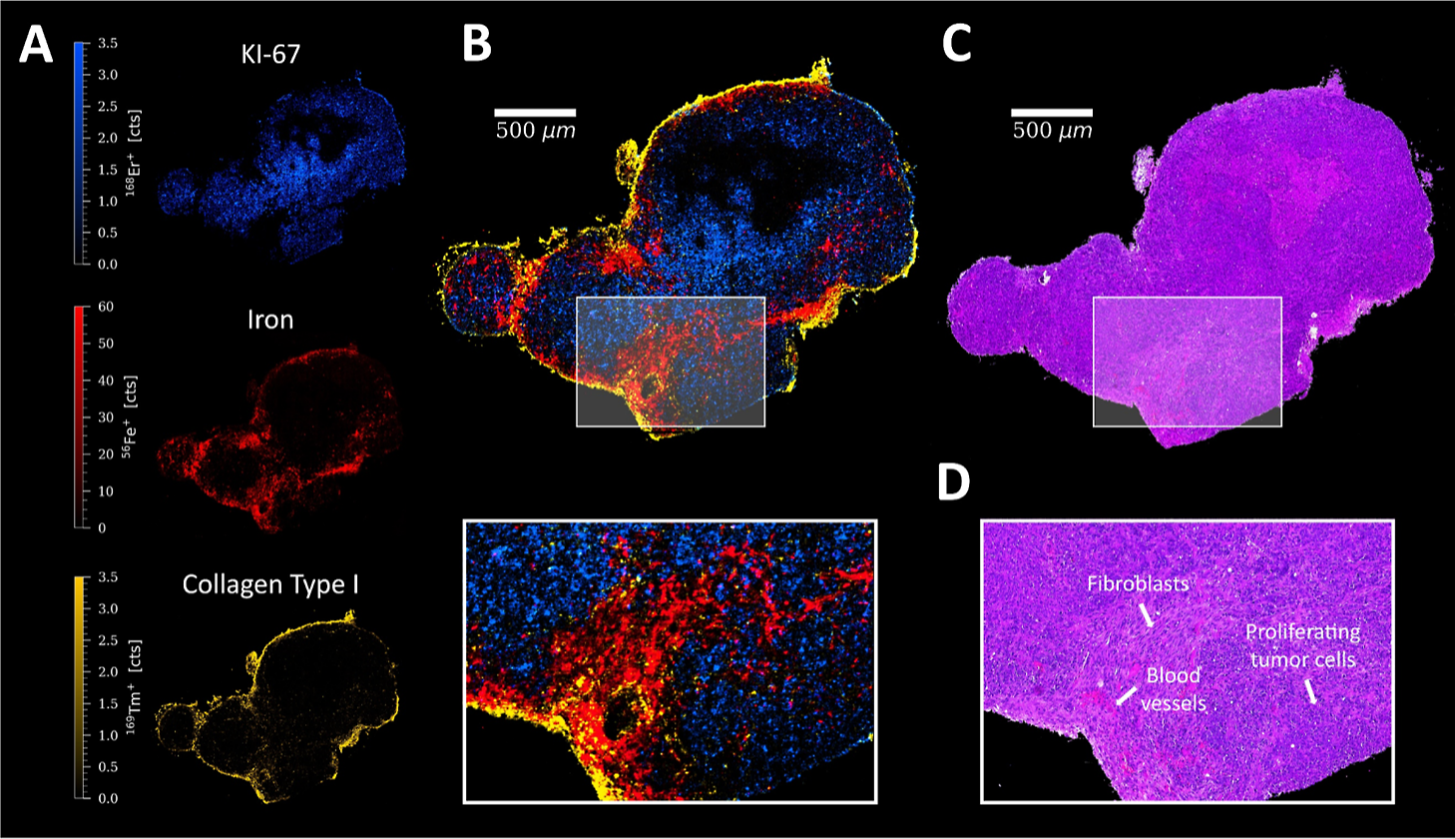
(A) Signal intensity maps and (B) signal overlay of KI-67 (blue), ^56^Fe^+^ (red), and Collagen Type I (yellow) in an HCT116 colon cancer tumor section of a mouse, with a ROI. (C) Corresponding H&E stain of a consecutive tumor section and (D) ROI with characteristic histological features.

For the characterization of the tumor microenvironment, the iron distribution can serve as a useful tool for visualizing blood vessels, which provides valuable information about tissue perfusion and potentially drug delivery and penetration into tumor tissue. An HCT116 colon cancer tumor section obtained from a human xenograft grown from CB-17/SCID mice was analyzed (Figure 7), which exhibited a low vascular density. A heterogeneous iron distribution was observed with irregular branching of blood vessels emanating from the outer layers and large zones of ischemia and necrosis, which is typical for rapid tumor growth.^47^ Collagen made up most of the outer tumor layer and acted as scaffold for the cancer cells (Figure 7).^48^ In the presence of iron, α-SMA can be found, revealing the cancer associated fibroblasts inside the tumor tissue (Figure S13).^49^ The majority of epithelial cells (indicated by pan-keratin) showed proliferation, whereas a large zone of necrosis was indicated by the absence of KI-67 and E-cadherin (Figures 7 and S11). Furthermore, this was confirmed by the corresponding H&E stain.^50^ Most of the DNA damage (pH2AX) corresponded to dead cells in this zone, but some of the surrounding proliferating cells were also affected (Figure S13).

## Conclusions

In this study, imaging mass cytometry was expanded toward simultaneous imaging of the cellular ionome. The results highlight the importance of evaluating multi-step sample preparation protocols for the analysis of endogenous elements in tissue samples by LA-ICPMS imaging. The elemental composition is already strongly influenced during tissue preparation (cryo-treatment, FFPE), an effect that is further enhanced by subsequent immunostaining procedures. While for selected elements such as Na, P, and Fe the qualitative tissue distribution remained intact after staining, quantification of endogenous elements was precluded. Tissue distributions of these elements with biological key functions were assessed at cellular resolution upon application of immunostaining procedures and allowed for linking ionome data to cell type/state and function. The added value of the validated wide mass range bioimaging strategy was emphasized using the prime example of preclinical in vivo models on cancer. Cell nuclei and parts of the cytoplasm could be visualized via the phosphorus signal, while sodium enabled the localization of renal corpuscles in the kidney and iron showed the red pulp with its marginal zones inside the spleen. This concept could be especially attractive in the context of disease progression, the evaluation of potential biomarkers, and the development of novel therapeutics. New embedding methods and sample preparation techniques with a reduced chemical background will be crucial to make full use of the potential of this promising technique.

## Methods

### Chemicals and Reagents

Ultrapure water (18.2 MΩ cm, ELGA Water purification system, Purelab Ultra MK 2, UK) was used for all dilutions and washing steps. A multi-element stock solution and single element standard solutions were purchased from Labkings (Hilversum, The Netherlands). Bovine serum albumin (lyophilized powder, BioReagent), Tris buffered saline (BioUltra), Triton X-100 (for molecular biology), m-xylene (anhydrous, ≥99%), and ethanol (absolute, EMSURE) were purchased from Sigma-Aldrich (Steinheim, Germany). The target retrieval solution was bought from Agilent Technologies (Waldbronn, Germany). Paraformaldehyde aqueous solution (Electron Microscopy grade, 16%) was obtained from Science Services (Munich, Germany) in form of sealed ampoules, to ensure fresh solutions in each staining procedure. The metal-conjugated antibodies used in this study (Table S3) and the Intercalator-Ir (Cell-ID, 125 μM) were purchased from Fluidigm (San Francisco, CA, USA). LA-ICP-TOFMS measurements were carried out in an ISO class 7 clean room. All cell culture media and reagents were purchased from Sigma-Aldrich (Vienna, Austria) and all plastic dishes, plates, and flasks from StarLab (Hamburg, Germany) unless stated otherwise.

### Cell Culture

The human colorectal cancer HCT116 cell line was kindly provided by Dr. Vogelstein from John Hopkins University, Baltimore. Cells were cultured in McCoy’s medium (M8403, Sigma-Aldrich, St. Louis, MO, USA) supplemented with 10% fetal calf serum (FCS; PAA, Linz, Austria) and 2 mM glutamine (Sigma-Aldrich, St. Louis, MO, USA). All cultures were grown under standard cell culture conditions and checked for *Mycoplasma* contamination.

### Animal Experiments

For in vivo experiments, 1 × 10^6^ HCT116 cells were injected subcutaneously (s.c.) in serum-free RPMI-medium (R6504, Sigma-Aldrich, St. Louis, MO, USA) into the right flank of 11-week-old male CB-17/SCID mice. Animals were kept in a pathogen-free environment and handled in a laminar airflow cabinet. The experiments were performed according to the regulations of the Ethics Committee for the Care and Use of Laboratory Animals at the Medical University Vienna (proposal number BMWF-66.009/0140-II/3b/2011), the U.S. Public Health Service Policy on Human Care and Use of Laboratory Animals, as well as the United Kingdom Coordinating Committee on Cancer Prevention Research’s Guidelines for the Welfare of Animals in Experimental Neoplasia. Tumors were palpable on day 7 following s.c. injection. Animals were controlled for symptoms of distress daily, and tumor size was assessed regularly by caliper measurement. Tumor volume was calculated using the formula (length × width^2^/2). On day 17, mice were sacrificed. Tumors and organs were formalin-fixed in 4% formaldehyde for 24 h (Carl Roth, #P087.3) and paraffin-embedded using a KOS machine (Milestone Medical, Sorisole, Italy).

### Histological Evaluations

For histological evaluation, embedded tumors and organs were cut into three consecutive 4 μm thick sections per set. Every first and third section was used for LA-ICP-TOFMS analysis. The second, middle section was used for H/E staining (Figure S14). Tissue was deparaffinized, rehydrated, and stained with hematoxylin/eosin (H/E) by routine procedures.

### Immunostaining of Cryo-Sections and FFPE Sections

The FFPE tumor tissue sections were deparaffinized by heating the slides in an oven for 1–2 h at 60 °C, followed by incubation with fresh xylene for 20 min. Descending grades of alcohol (100–70% EtOH) were used for re-hydration. After washing the slides with ultrapure water, heat-induced antigen retrieval was performed at 96 °C for 30 min using an antigen retrieval solution (Tris–EDTA, pH = 9). The slides were carefully cooled down and washed with ultrapure water and TBS. Cryo-sections were first fixed for 30 min with 4% PFA in TBS and then rinsed 3 times with TBS. Both types of sections were incubated with 3% BSA in TBS for 45 min at RT to block unspecific binding sites. The sections were then incubated with a cocktail of metal-tagged antibodies in a hydration chamber overnight at 4 °C. A summary of the metal-conjugated antibodies employed in this study can be found in Table S3. The antibody solution was prepared by adding small amounts of each antibody (1:50–1:200 dilutions of the respective antibodies) to 0.5% BSA in TBS. In order to avoid the formation of aggregates, the antibodies were centrifuged beforehand at 13,000 g for 2 min. For cell permeabilization, the slides were incubated in 0.2% Triton X-100 and washed afterward with TBS. A Cell-ID Intercalator-Ir (125 μM, Fluidigm, San Francisco, CA, USA) was used to stain the tissue sections, by adding the solution (0.30 μM) on the sections and incubating them for 30 min at RT in a hydration chamber. Finally, the slides were repeatedly washed with ultrapure water and left to air-dry until LA-ICP-TOFMS analysis.

### Calibration Standards for LA-ICP-TOFMS Analysis

Quantification was performed by LA-ICP-TOFMS using gelatin-microdroplets, as described previously.^35^ For this purpose, liquid multi-element standard solutions were prepared gravimetrically from commercial standard stock solutions in 1% (v/v) HNO_3_. Gelatin stock solution (10%, w/w) was added to reach a final concentration of 1% (w/w) gelatin. The resulting solutions were transferred into wells of a 384 well plate, which serves as the sample source of a micro-spotter system. A CellenONE X1 micro-spotter (Cellenion, Lyon, France) was used to generate arrays of the gelatin micro-droplet standards onto glass slides with a droplet size of 400 ± 5 pL resulting in droplet sizes of around 100 μm in diameter. The size of the droplets was evaluated by the software of the instrument and was used for normalization to establish absolute elemental quantities within the droplets. The entire micro-droplets were quantitatively and selectively ablated, and multi-element analysis was performed by LA-ICP-TOFMS.

### LA-ICP-TOFMS Analysis

An Iridia 193 nm laser ablation (LA) system (Teledyne Photon Machines, Bozeman, MT, USA) was coupled to an *icp*TOF 2R (TOFWERK AG, Thun, Switzerland) ICP-TOFMS instrument. The LA system was equipped with an ultrafast low-dispersion cell^51^ in a Cobalt ablation chamber and coupled with the aerosol rapid introduction system (ARIS) to the ICP-TOFMS. An Ar make-up gas flow (∼0.90 L min^–1^) was introduced through the low-dispersion mixing bulb of the ARIS into the He carrier gas flow (0.60 L min^–1^) before entering the plasma. Daily tuning of the LA and ICP-TOFMS settings was performed using NIST SRM612 glass certified reference material (National Institute for Standards and Technology, Gaithersburg, MD, USA). Optimization was based on high intensities for ^24^Mg^+^, ^59^Co^+^, ^115^In^+^, and ^238^U^+^, low oxide formation based on the ^238^U^16^O^+^/^238^U^+^ ratio (<2%) and low elemental fractionation based on the ^238^U^+^/^232^Th^+^ ratio (∼1). Daily optimization included to aim at a low aerosol dispersion characterized by the pulse response duration for ^238^U^+^ based on the FW0.01 M criterion, that is, the full peak width of the ^238^U^+^ signal response obtained upon a single laser shot, at 1% of the height of the maximum signal intensity. Laser ablation sampling was performed in fixed dosage mode 2, at a repetition rate of 200 Hz and using a 5 μm spot size (square) with an interspacing of 2.5 μm between the lines resulting in a pixel size of 2.5 μm × 2.5 μm. Selective ablation of the samples was achieved by selecting an energy density below the ablation threshold of glass and above the ablation threshold of the samples.^52^ Gelatin micro-droplets, organs, and tumor sections were removed quantitatively using a fluence of 0.60 and 0.80 J cm^–2^, respectively.

The *icp*TOF 2R ICP-TOFMS instrument has a specified mass resolution (*R* = *m*/Δ*m*) of 6000 (full width half-maximum definition) and allows for the analysis of ions from *m/z* = 14–256. The integration and read-out rate match the LA repetition rate. The instrument was equipped with a torch injector of 2.5 mm inner diameter and nickel sample and skimmer cones with a skimmer cone insert of 2.8 mm in diameter. A radio frequency power of 1440 W, an auxiliary Ar gas flow rate of ∼0.80 L min^–1^, and a plasma Ar gas flow rate of 14 L min^–1^ were used. For all measurements, the collision cell technology (CCT) mode was used, where the collision cell was pressurized with a mixture of H_2_/He gas [93% He (v/v), 7% H_2_ (v/v)] with an optimized flow rate of 4.2 mL min^–1^. The following CCT parameters were used: CCT focus lens: −6.3 V, CCT entry lens: −150 V, CCT mass: 261 V, CCT bias: −1 V, CCT exit lens: −90 V. Instrumental parameters for LA-ICP-TOFMS measurements in CCT mode are summarized in Table S4.

### Data Acquisition and Processing of LA-ICP-TOFMS Data

LA-ICP-TOFMS data were recorded using TofPilot 2.10.3.0 (TOFWERK AG, Thun, Switzerland) and saved in the open-source hierarchical data format (HDF5, www.hdfgroup.org). Post-acquisition data processing was performed with Tofware v3.2.2.1 (TOFWERK AG, Thun, Switzerland), which is used as an add-on on IgorPro (Wavemetric Inc., Oregon, USA). The data processing included (1) drift correction of the mass peak position in the spectra over time via time-dependent mass calibration, (2) determining the peak shape, and (3) fitting and subtracting the mass spectral baseline. Data was further processed with HDIP version 1.6.6.d44415 × 105 (Teledyne Photon Machines, Bozeman, MT, USA). An integrated script was used to automatically process the files generated by Tofware and to generate 2D elemental distribution maps.

### Statistical Methods

Elemental channel correlations were calculated using Spearman’s rank correlation coefficient, while the SSIM was used to score image similarity. Correlation matrices were created using the Python programming language v3.9, specifically by use of the following libraries: NumPy v1.23.0; pandas v1.4.3; Seaborn v0.11.2; and scikit-learn v1.1.1.

## Supporting Information Available

Elemental background of staining chemicals, elemental recoveries after labeling, list of metal-conjugated antibodies, instrumental parameters, cell morphology for FFPE and cryo-section, elemental distributions after labeling (Fe, Cu, and Zn), image comparison using Structural Similarity Index, Spearmańs correlation matrix for selected regions of interest, signal intensity maps of metal-conjugated antibodies for organs and tumor, comparison of phosphorus and iridium signal for cell nuclei in liver, kidney structure using E-Cadherin, closeup of renal corpuscle, infiltrating tumor cells in lung, and hematoxylin & eosin stains of analyzed tissues (PDF)
