# Supplementary material for: Multiparametric Tissue Characterization Utilizing the Cellular Metallome and Immuno-Mass Spectrometry Imaging
Source: JACS Au. 2023 Feb 8;3(2):419–28. doi: 10.1021/jacsau.2c00571 (PMC9975846; doi:10.1021/jacsau.2c00571)
Supplement: Supplementary file 1 — au2c00571_si_001.pdf [file au2c00571_si_001.pdf]

Supporting Information

Multiparametric tissue characterization utilizing the cellular metallome and immuno-mass spectrometry imaging

Martin Schaier[1,2], Sarah Theiner[1,*], Dina Baier[3,4], Gabriel Braun[1,2], Walter Berger[4], Gunda Koellensperger[1,*]

[1] Institute of Analytical Chemistry, Faculty of Chemistry, University of Vienna, Waehringer Strasse 38, 1090 Vienna, Austria

[2] Vienna Doctoral School in Chemistry (DoSChem), University of Vienna, Währinger Strasse 42, 1090 Vienna, Austria.

[3] Institute of Inorganic Chemistry, Faculty of Chemistry, University of Vienna, Waehringer Strasse 42, 1090 Vienna, Austria

[4] Institute of Cancer Research and Comprehensive Cancer Center, Medical University of Vienna, Borschkegasse 8A, 1090 Vienna, Austria

* Corresponding authors:

Gunda Koellensperger

Institute of Analytical Chemistry, Waehringer Strasse 38, 1090 Vienna, Austria

Tel: +43-1-4277-52303, Email: gunda.koellensperger@univie.ac.at

Sarah Theiner

Institute of Analytical Chemistry, Waehringer Strasse 38, 1090 Vienna, Austria

Tel: +43-1-4277-52384, Email: sarah.theiner@univie.ac.at

Table S1. Elemental background of chemicals used during immunostaining. Solutions were digested and analyzed using direct-infusion ICP-MS.

	Elemental background [$\mu g\ L^{-1}$]				
	**Bovine serum albumin (10%)**	**Paraformaldehyde**	**Antigen retrieval solution (pH 9)**	**TRIS buffered saline**	**Ethanol**
**Na**	1855585	1781256	2250378	1770497	763
**Mg**	2381	26	<LOQ	<LOQ	<LOQ
**P**	2942	<LOQ	110	229	117
**K**	4337	140	<LOQ	136	<LOQ
**Ca**	10662	6	<LOQ	292	203
**Fe**	2184	81	85	87	104
**Cu**	1193	6	4	28	2
**Zn**	502	1645.	20	51	17

* <LOQ = The obtained concentration was under the limit of quantification.

Table S2. Recoveries of endogenous elements after the labeling procedure with metal-conjugated antibodies. Consecutive cryo-sections and FFPE sections of tumor tissue before and after labeling were analyzed by LA-ICP-TOFMS.

**Element**	**Recovery [%] Cryo-section**	**Recovery [%] FFPE**
**Na**	44	140
**Mg**	1	5
**P**	69	63
**K**	3	<LOQ*
**Ca**	24	14
**Fe**	79	150
**Cu**	7400	5800
**Zn**	13	5

*K concentration was under the limit of quantification.

Table S3. Panel of metal-conjugated antibodies used for immunostaining.

Antibody target	Clone	Metal tag	Catalog number	Function
**Structural/ environmental marker**				
α-SMA	1A4	^{141}Pr	3141017D	Smooth muscle/ myofibroblasts
Vimentin	D21H3	^{143}Nd	3143027D	Fibroblasts/ blood vessels
Pan-Keratin	C11	^{148}Nd	3148020D	Epithelial cells
E-Cadherin	24E10	^{158}Gd	3158029D	Epithelial cell membrane
Collagen Type I	Polyclonal	^{169}Tm	3169023D	Extracellular matrix
**Immune cell marker**				
CD19	6OMP31	^{142}Nd	3142014D	B cells
CD11b	EPR1344	^{149}Sm	3149028D	Myeloid cells
CD11c	3.9	^{154}Sm	3154026D	Dendritic cells
CD86	IT2.2	^{156}Gd	3156035D	M1 macrophages
Arginase-1	D4E3M	^{164}Dy	3164027D	M2 macrophages
CD45RA	HI100	^{166}Er	3166031D	Naive T cells
CD3	Polyclonal. C-Terminal	^{170}Er	3170019D	Pan T cell
CD45RO	UCHL1	^{173}Yb	3173016D	Memory T-cells
**Other marker**				
BCL-2	EPR17509	^{146}Nd	3146019D	Apoptosis suppression
CD44	IM7	^{153}Eu	3153029D	Tumor/EMT
pH2AX	S139	^{165}Ho	3165036D	DNA damage
KI-67	B56	^{168}Er	3168022D	Proliferation
Caspase 3	5A1E	^{172}Yb	3172027D	Apoptosis

Table S4. LA-ICP-TOFMS parameters.

**ICP-TOFMS**	
RF Power [W]	1440
Sampling depth [mm]	3.50
Cone materials	Ni
Plasma gas flow [L min^{-1}]	14.0
Auxiliary gas flow [L min^{-1}]	0.80
Nebulizer gas flow [L min^{-1}]	1.00
Measurement mode	Collision cell technology (CCT)
CCT gas	93% He *(v/v)*, 7% H_2 *(v/v)*
CCT gas flow [mL min^{-1}]	4.20
m/z range	14-256
**Laser ablation**	
Spot size	5 µm (square)
Interspacing	2.5 µm
Repetition rate	200 Hz
Dosage	2
Fluence	0.60 - 0.80 J cm^{-2}

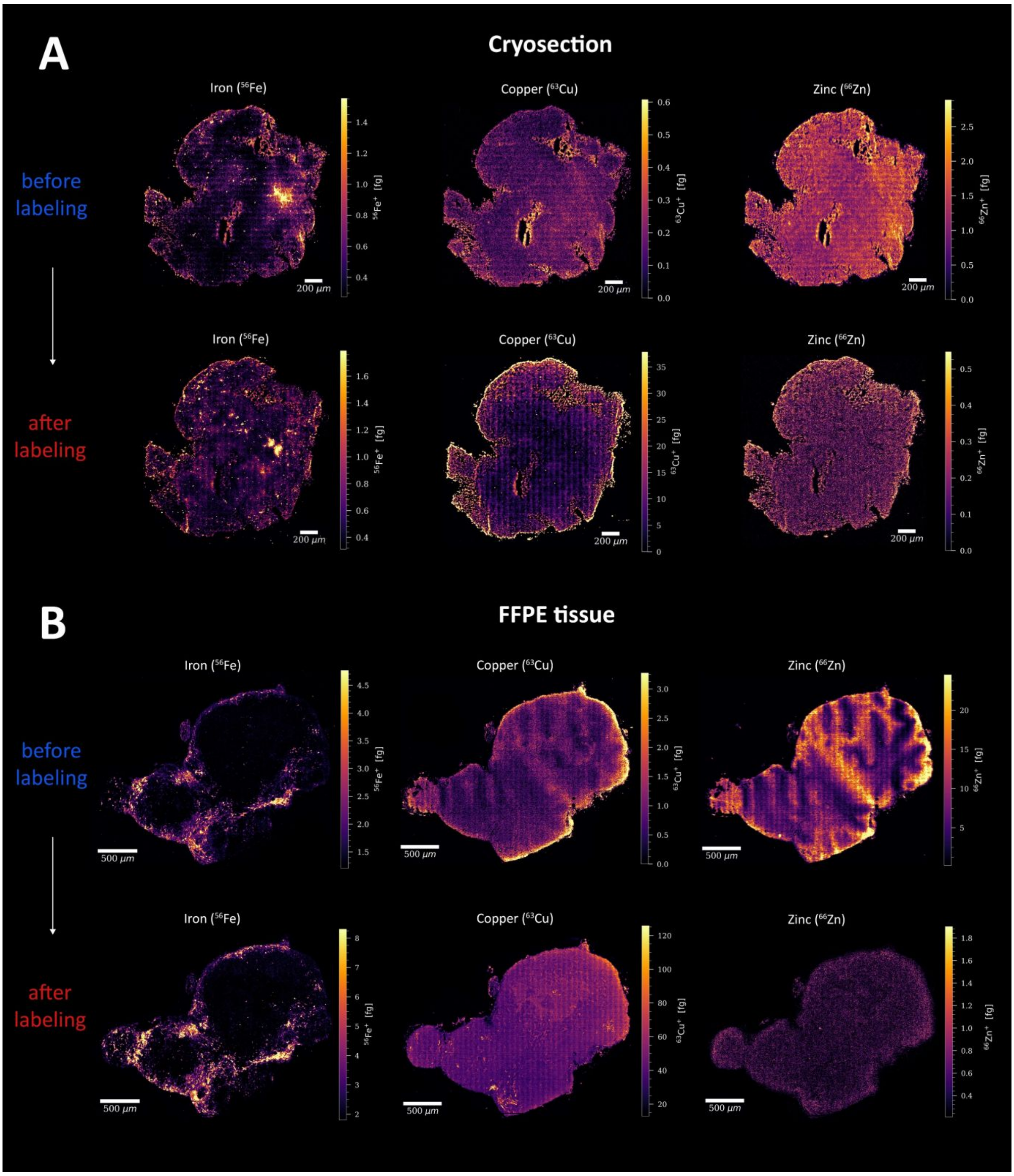

Fig. S1. The signal intensity maps of the endogenous elements $^{56}Fe^{+}$, $^{63}Cu^{+}$ and $^{66}Zn^{+}$ in tumor tissue before and after the metal-conjugated antibody staining procedure, using cryo-sections (A) and FFPE sections (B).

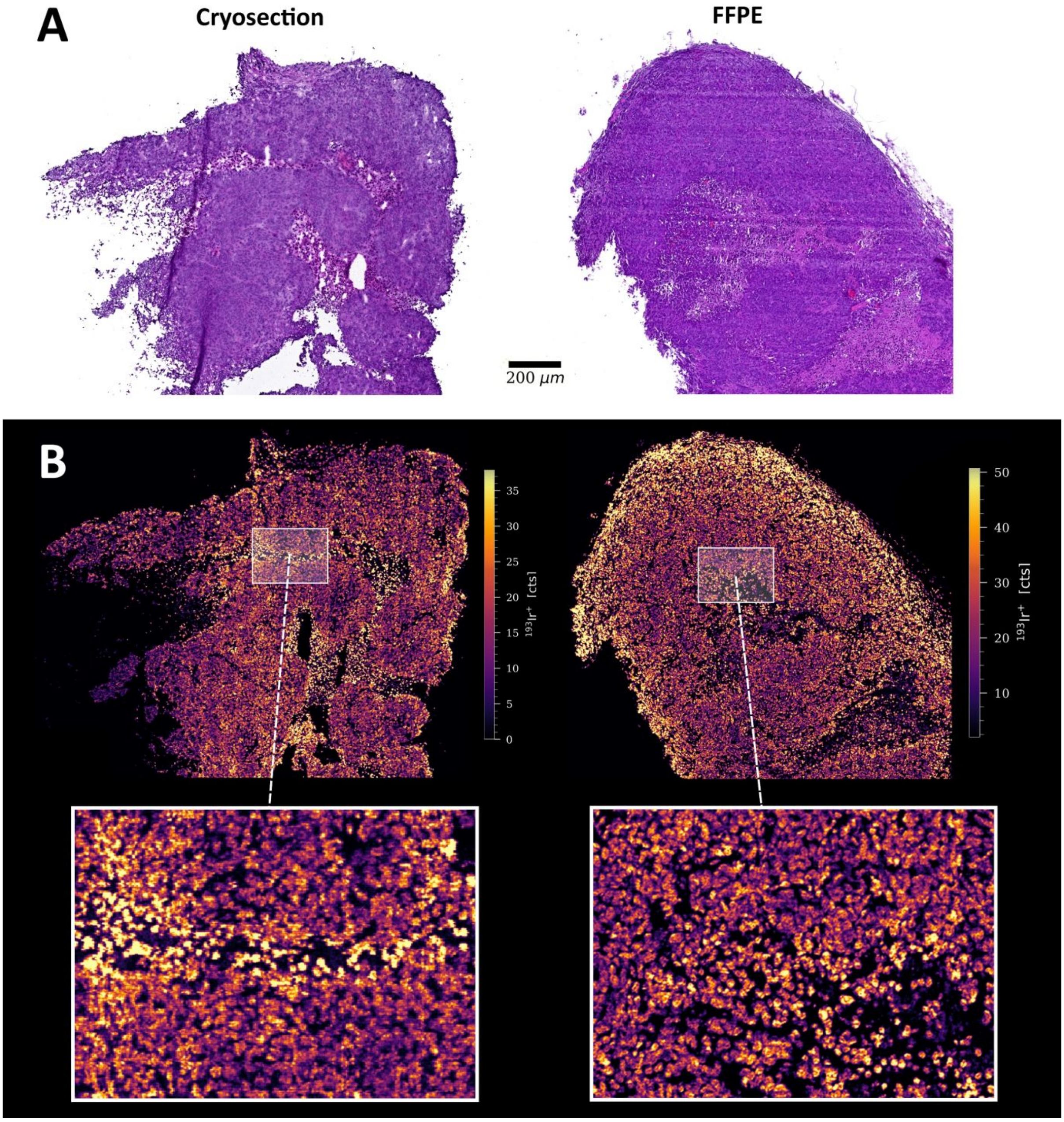

Fig. S2. Comparison of the tissue quality for the two embedding methods (cryo-sectioning and FFPE), using HCT116 tumor sections. In the H&E stains. (A) the cryo-section showed cutting artifacts (e.g. tissue folding), while the FFPE section showed an intact structure. Signal intensity maps of an Ir-intercalator (B) measured by LA-ICP-TOFMS showed a better preserved cell morphology for FFPE tissue.

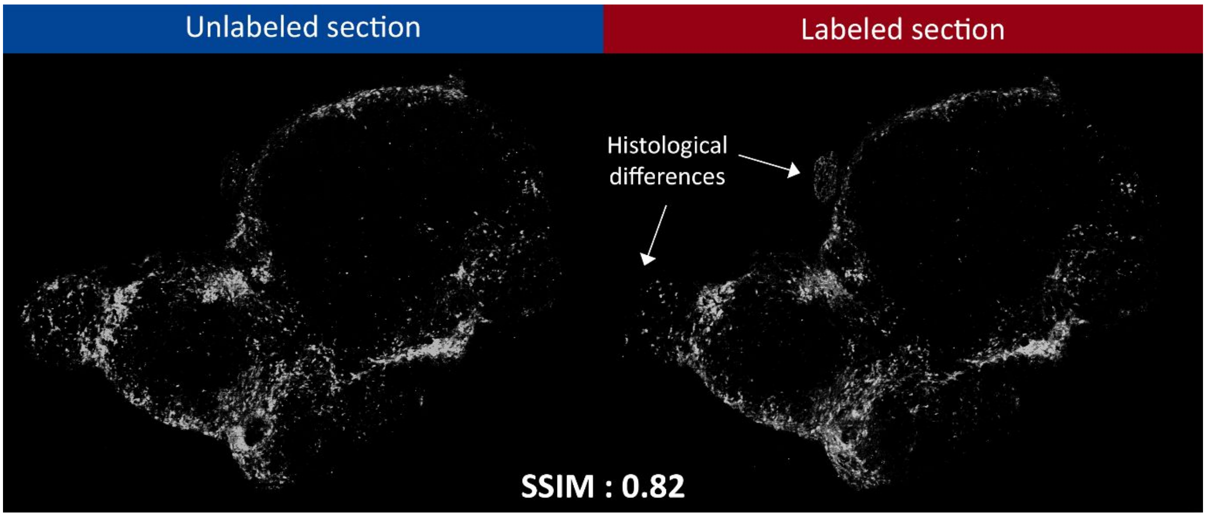

Fig. S3. Comparison of iron signal intensity maps from two consecutive mouse tumor sections using the Structural Similarity Index (SSIM), where a value of 1 indicates strong and of -1 weak similarity. The images are not identical due to histological differences caused by consecutive sectioning of the tissue.

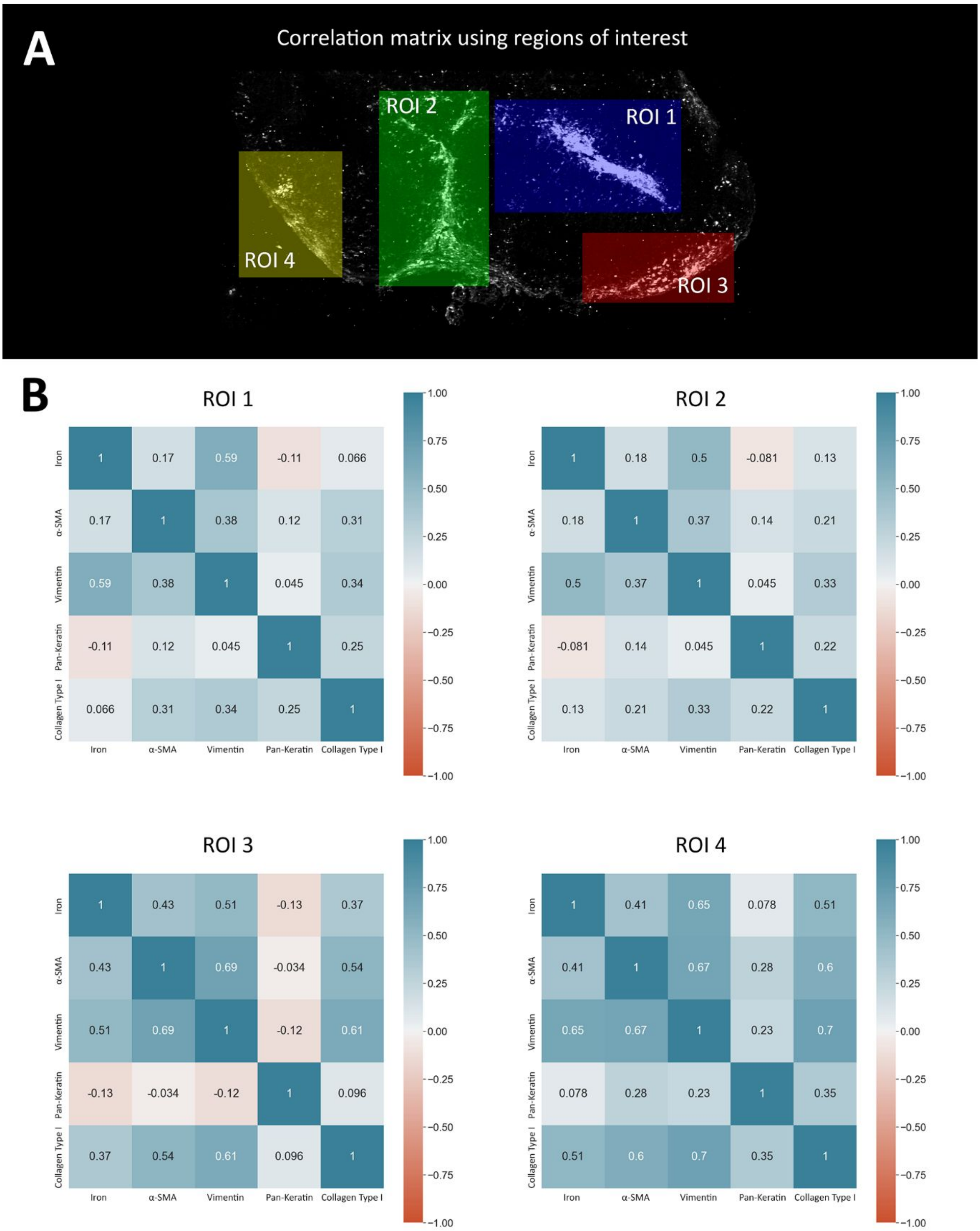

Fig. S4. (A) Different ROIs were selected for the iron signal intensity maps measured in tumor tissue. (B) Co-localization in these areas was assessed using correlation matrices that included four metal-conjugated antibodies. The values shown indicate Pearson correlation coefficient (0.5-1: strong positive, 0.3-0.5: moderate positive, 0-0.3: weak positive).

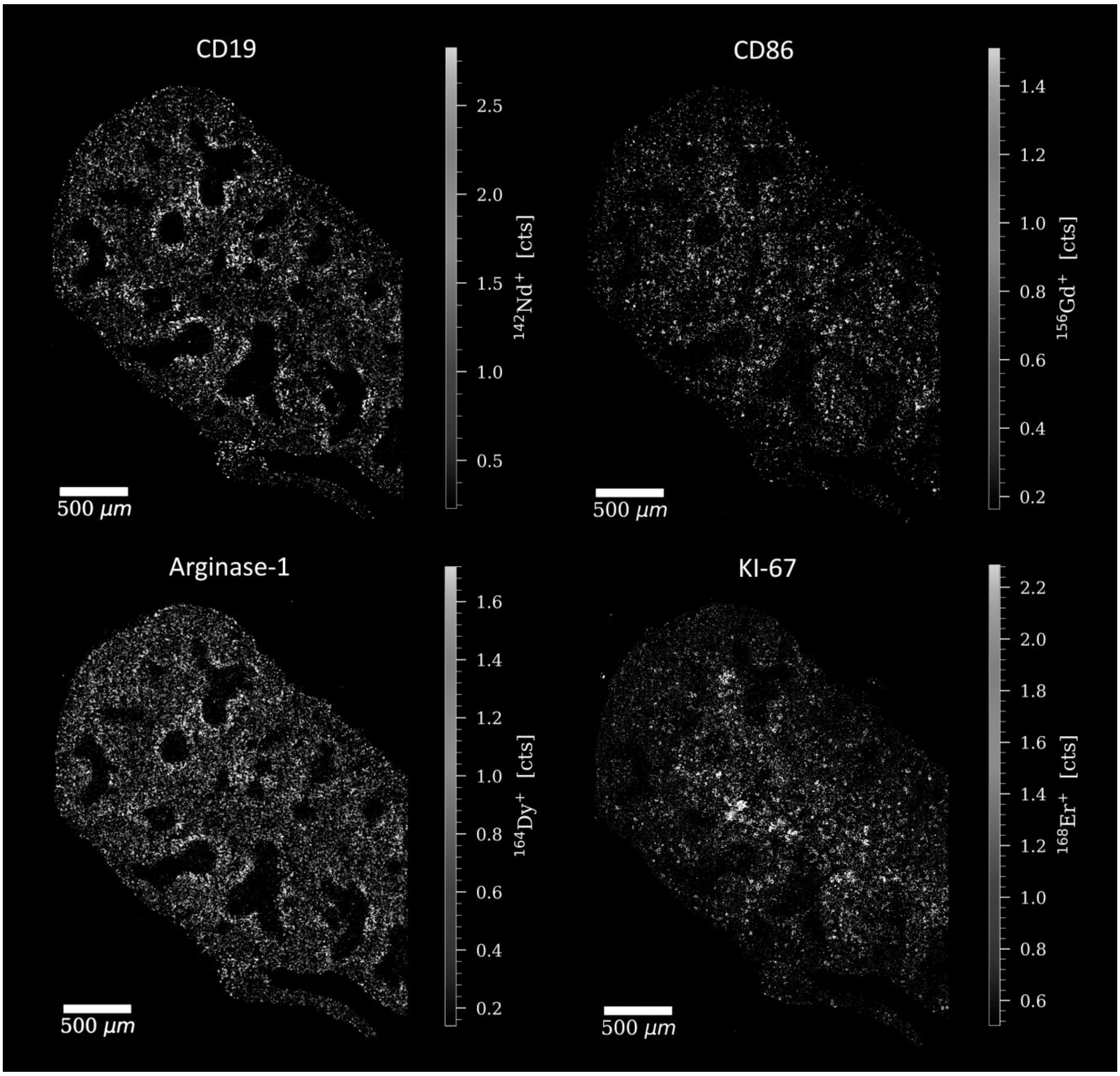

Fig. S5. Signal intensity maps for different metal-conjugated antibodies in mouse spleen.

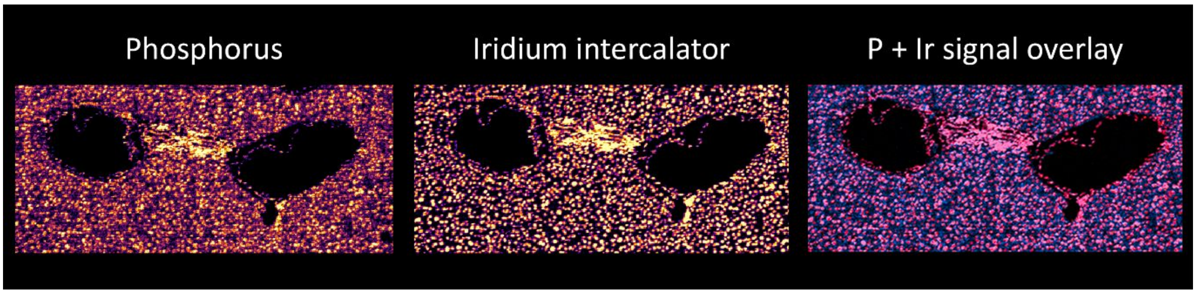

Fig. S6. Comparison of signal intensity maps for phosphorus and iridium in mouse liver. The iridium intercalator shows a specific signal for the cell nuclei, while phosphorus also gives

information about the cytoplasm at a lower intensity. This is most evident in the P/Ir overlay showing the additional phosphorus signal in blue.

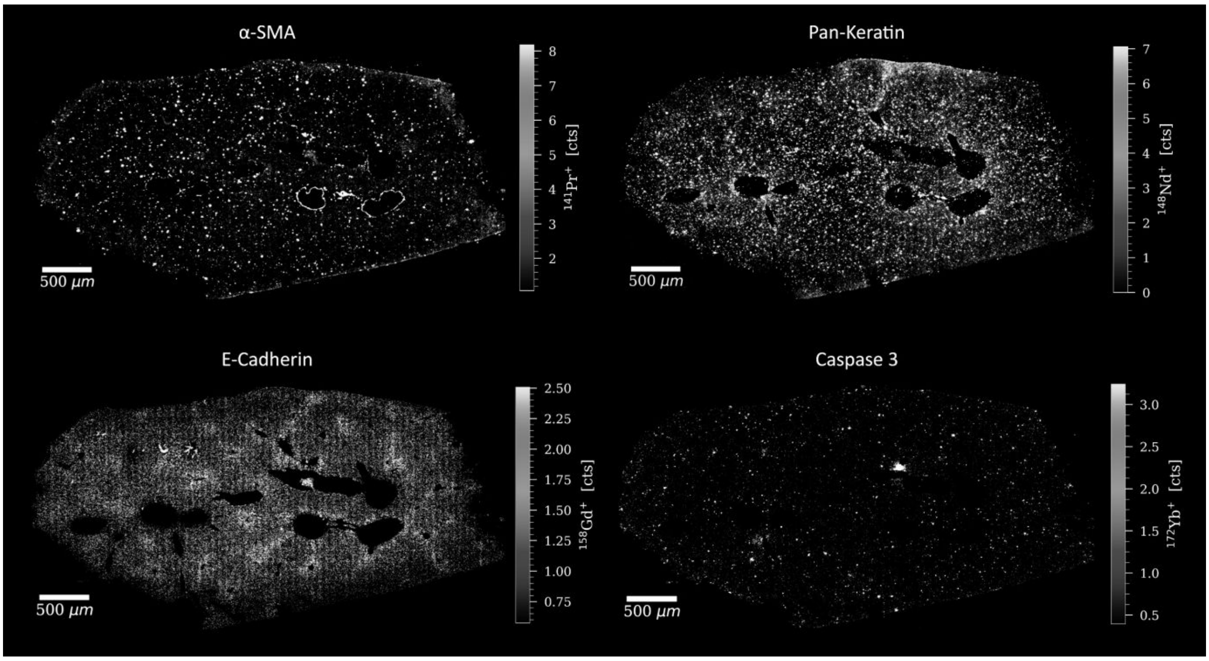

Fig. S7. Signal intensity maps for different metal-conjugated antibodies in mouse liver.

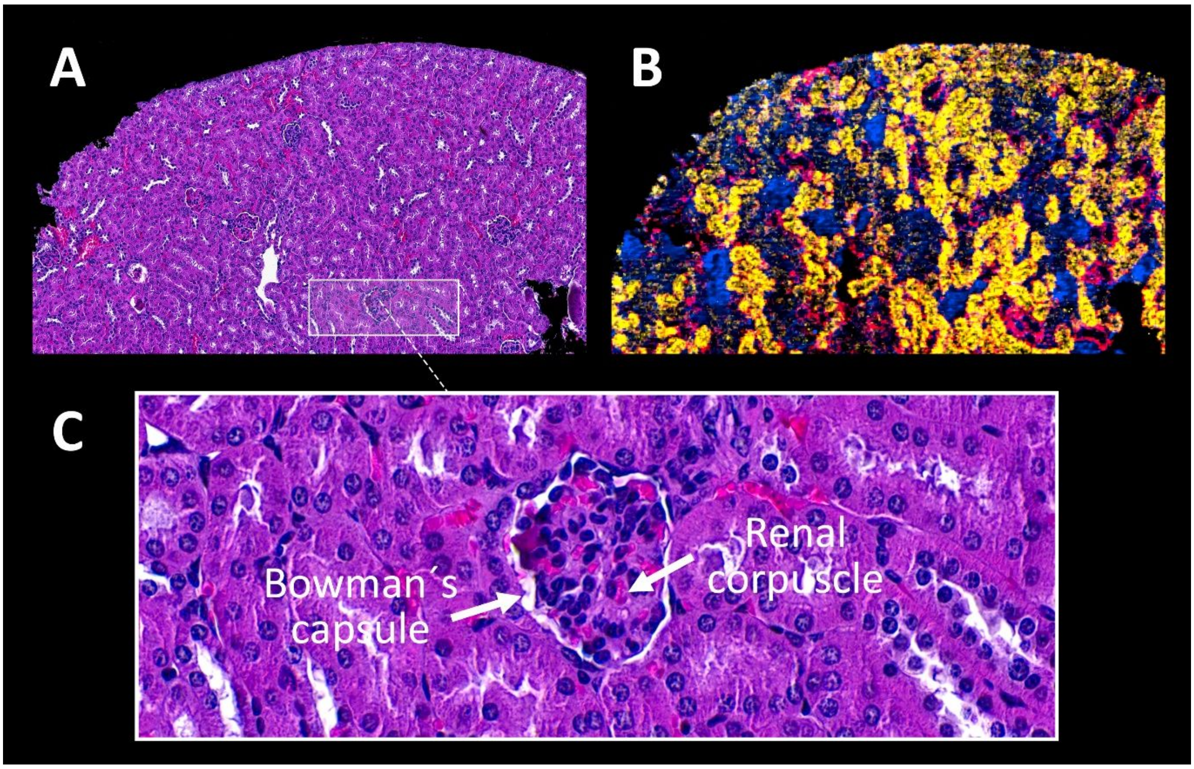

Fig. S8. (A) Renal corpuscle visible in the H&E stain can be correlated with (B) sodium hotspots (blue). A higher magnification (C) shows the corpuscle surrounded by the Bowman´s capsule in more detail.

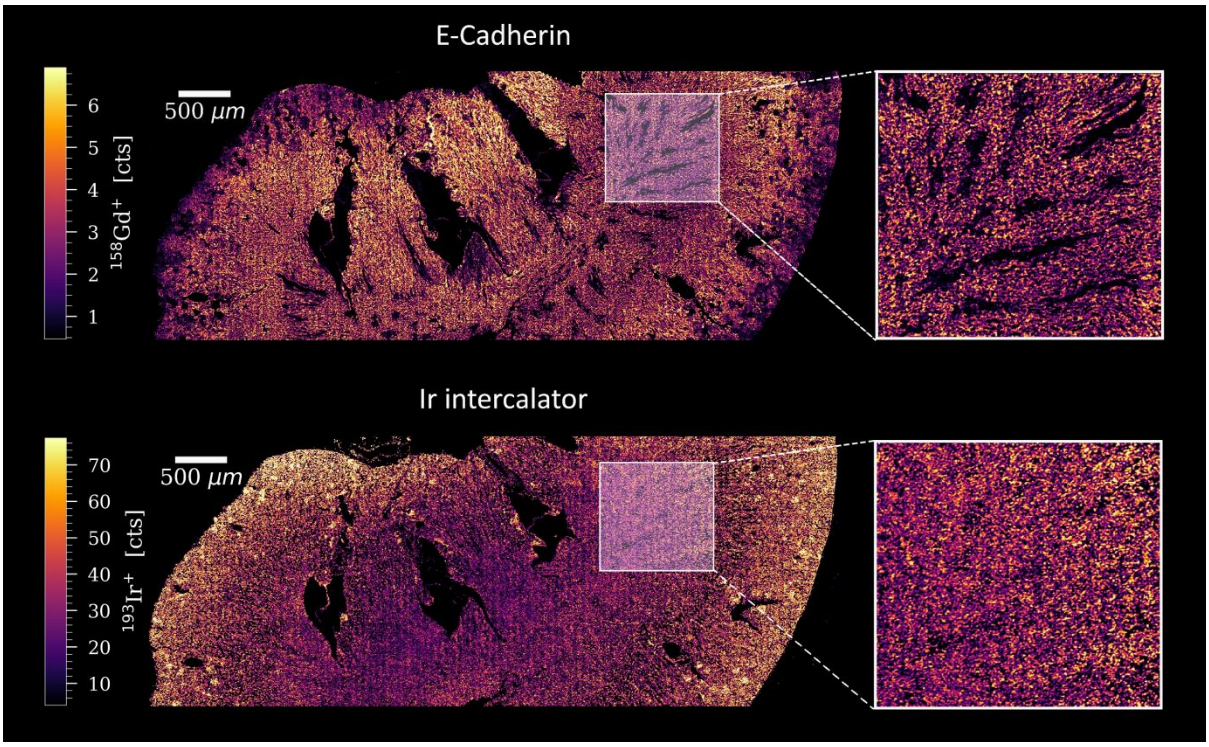

Fig. S9. Comparison of the signal intensity maps of E-cadherin and the iridium intercalator. Since iridium is intended for marking cell nuclei, it shows the structure in the kidney relatively imprecisely, as the renal cells contain several cell nuclei. E-cadherin, a membrane marker of epithelial cells, on the other hand, is better suited to depict individual cells and the tissue structure, as seen in the closeup.

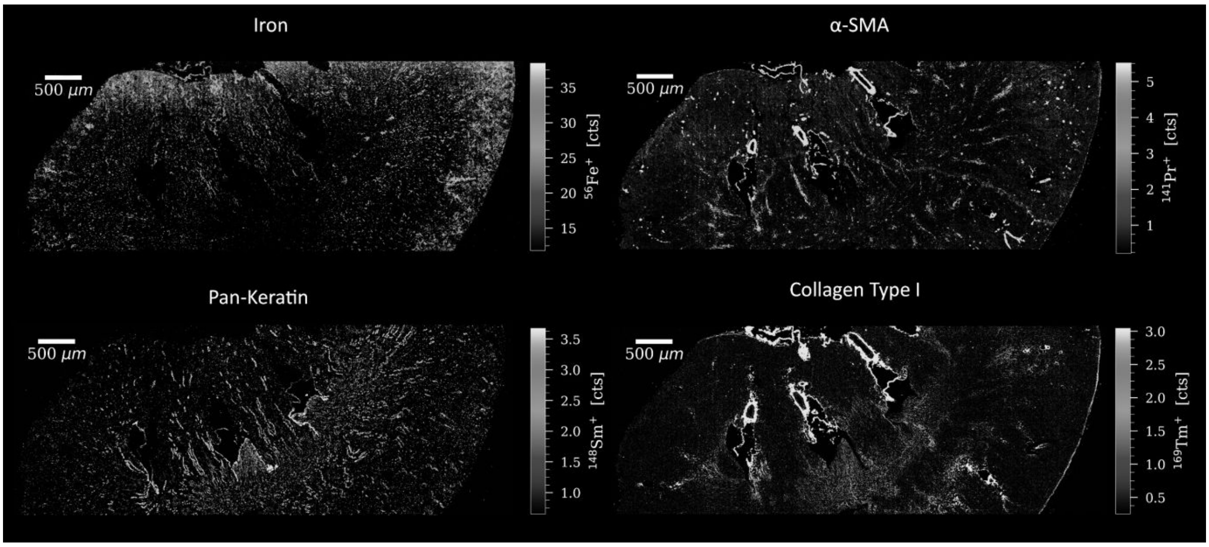

Fig. S10. Signal intensity maps for different metal-conjugated antibodies in mouse kidney.

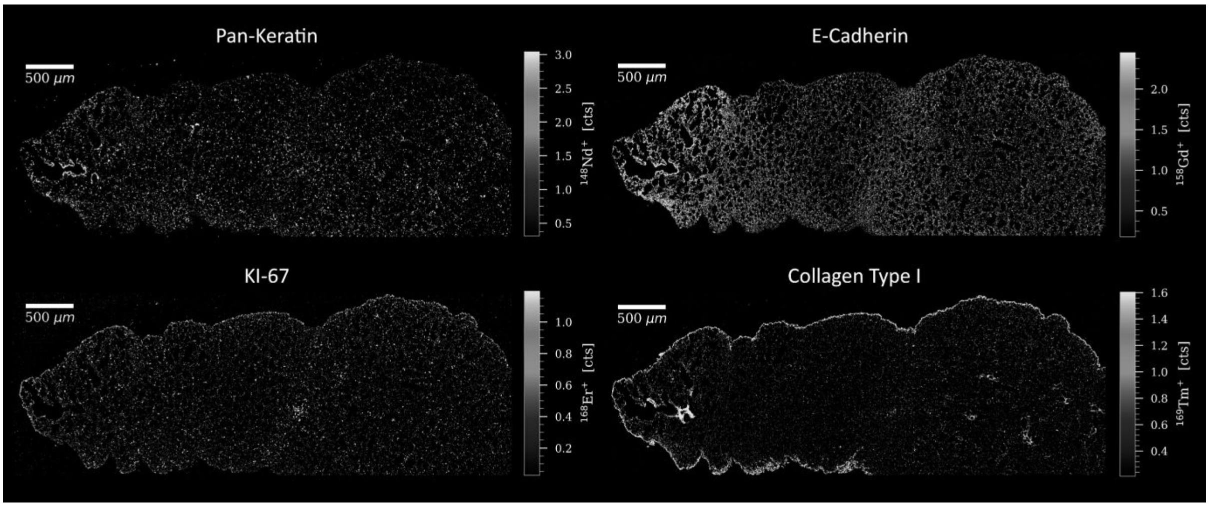

Fig. S11. Signal intensity maps for different metal-conjugated antibodies in mouse lung.

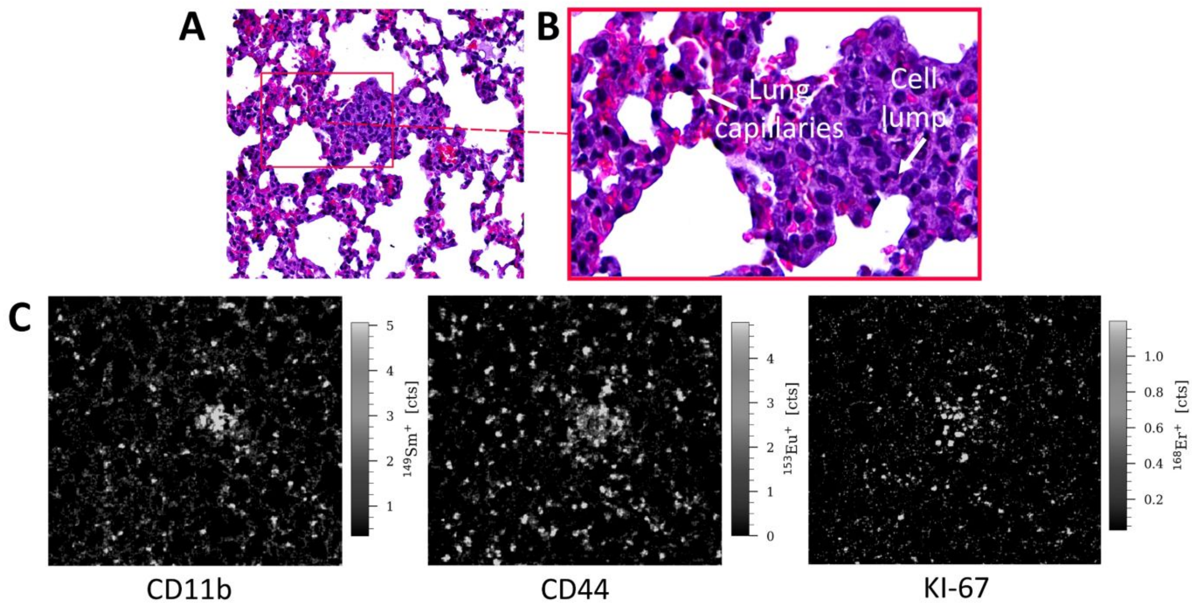

Fig. S12. (A) A region of interest with (B) a close up of the mouse lung section stained with H&E. (C) A spot with high accumulation of different antibodies corresponding to the ROI of (A) can be seen, which indicates infiltrating tumor cells.

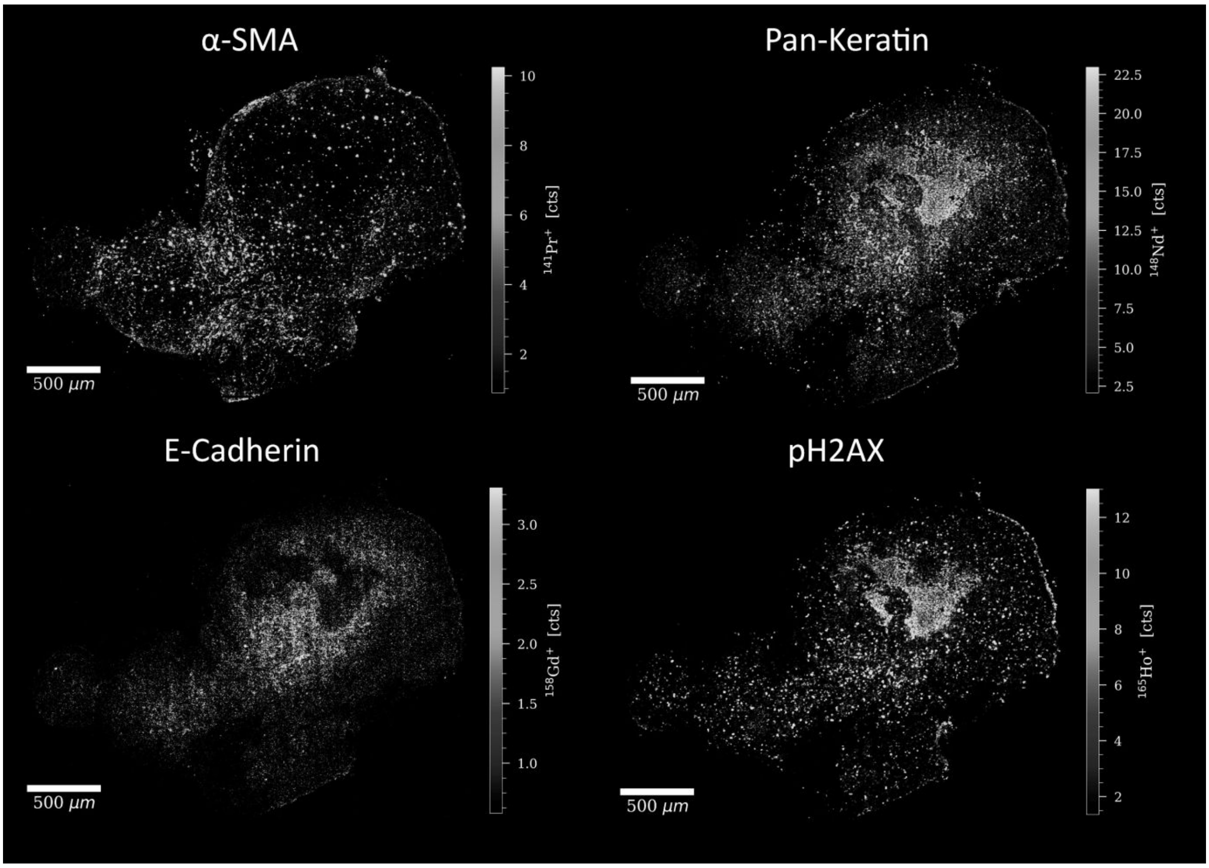

Fig. S13. Signal intensity maps for different metal-conjugated antibodies in an HCT116 xenograft tumor.

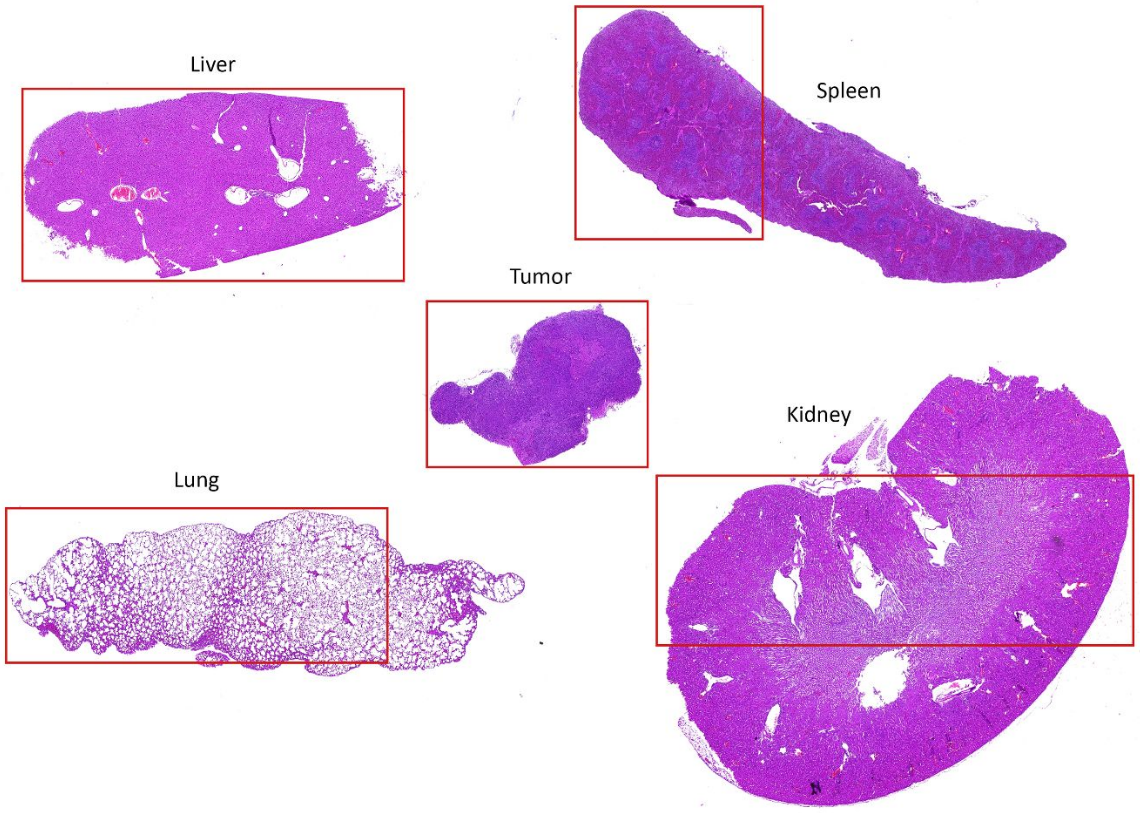

Fig. S14. Microscopic images of H&E-stained tissue sections used in this study. Different areas of consecutive sections were analyzed using iMSI by LA-ICP-TOFMS (marked red).
